# Dépistage du risque podologique chez les diabétiques de type 2 à Antananarivo

**DOI:** 10.11604/pamj.2017.27.213.11311

**Published:** 2017-07-20

**Authors:** Sitraka Angelo Raharinavalona, Haritsiky Robertini Ramalanjaona, Nalisoa Andrianera, Andrinirina Dave Patrick Rakotomalala, George Ramahandridona

**Affiliations:** 1 Service d’Endocrinologie, Hôpital Joseph Raseta de Befelatanana, Antananarivo, Madagascar; 2 Clinique et Centre d’Education de Diabète de l’AMADIA Madagascar Diabetes Association - Faravohitra Antananarivo, Madagascar

**Keywords:** Dépistage, éducation, facteurs de risque, prévention, pied diabétique, Screening, education, risk factors, prevention, diabetic foot disease

## Abstract

Le pied diabétique pèse lourdement sur la santé des diabétiques, étant responsable d’une grande morbidité et de graves incapacités. Leur prévention reste incontournable. Notre étude vise à réduire le taux d’amputation chez les diabétiques. Nous avons fait une étude rétrospective descriptive, transversale et multicentrique du dépistage du risque podologique chez les diabétiques de type 2, au service d’Endocrinologie de l’Hôpital Universitaire de Befelatanana et à la Clinique AMADIA, Faravohitra, sur une période de 6 mois. L’âge moyen de nos patients était de 54,43 ans. Le genre masculin prédominait (60%). Leur diabète évoluait, en moyenne, depuis 7,35 ans. Les antécédents les plus observés étaient le tabagisme et les ulcérations chroniques du pied. Plus de la moitié de nos patients avaient un diabète déséquilibré, une microalbuminurie et une rétinopathie. Nous avons enregistré 56,50% de patients conscients du risque mais moins de 46% donnant des exemples précis sur les conduites adaptées. Seulement 13,33% de nos patients bénéficiaient d’un examen des pieds avant la présente étude. Leur risque podologique était très élevé. Les facteurs prédictifs d’ulcération identifiée étaient le tabagisme, l’antécédent d’ulcération chronique des membres inférieurs, le mauvais équilibre du diabète et l’existence d’une déformation du pied. La stratégie de prévention efficace des amputations doit comporter le dépistage et l’identification de la population à risque d’ulcération.

## Abstract (en)

Diabetic foot disease heavily weighs upon diabetics, being a significant cause of morbidity and severe disability. Their prevention is indispensable. Our study aimed to reduce the rate of amputations in diabetics. We conducted a retrospective, descriptive, cross-sectional, multicenter study of podiatric risk screening in patients with type 2 diabetes in the Endocrinology Department at the University Hospital of Befelatanana and at the Amadia Clinic, Faravohitra, over a period of 6 months. The average age of our patients was 54.43 years. Male sex predominated (60%). The patients suffered from diabetes which had evolved, on average, over a period of 7.35 years. The most common patient’s history was smoking and chronic ulcerations of the foot. More than half of patients had an imbalance of diabetes, microalbuminuria and retinopathy. 56.50% of patients were aware of their risk but less than 46% of patients gave specific examples of appropriate behaviour. Only 13.33% of patients had undergone foot examination before this study. Their podiatric risk was very high. Predictive factors for ulceration included smoking, a history of chronic ulcer of the lower limbs, an imbalance of diabetes and foot deformity. Effective prevention strategies for amputations should include the screening and the identification of population at risk of ulceration.

## Introduction

Le diabète sucré est un groupe de maladies métaboliques caractérisé par une hyperglycémie chronique résultant d’un défaut de sécrétion, ou d´action d’insuline ou des deux à la fois. Cette hyperglycémie chronique, à long terme, entraine des dommages, un dysfonctionnement, et une défaillance de différents organes [1]. Particulièrement, au niveau des pieds, le diabète entraine des lésions groupées sous le terme « pied diabétique ». Il se définit par l’ensemble de troubles trophiques consécutives à des atteintes nerveuses, artérielles et souvent infectieuses survenant sur le pied d’un diabétique [2]. Le « pied diabétique », responsable d’une grande morbidité invalidante, pèse lourdement sur la santé des diabétiques, devenant un véritable problème de santé publique [3]. Dans le monde, une amputation au niveau des membres inférieurs, inhérente au diabète serait réalisée toutes les 30 secondes [4]. Une plaie mineure du pied serait à l’origine de 85% des amputations et quatre plaies sur cinq ont une origine externe identifiable unique et a priori évitable [5]. La gradation du risque de plaie du pied est encore insuffisante chez le patient diabétique [6]. Or Selon l’American Diabetes Association (ADA), il est recommandé d’effectuer un examen annuel complet des pieds pour identifier les facteurs de risque prédictifs des plaies et des amputations chez tous les diabétiques, groupés sous le terme de risque podologique [7]. Si dans les pays développés, beaucoup d’études ont été faites à propos du risque podologique. Pour le continent Africain, en particulier à Madagascar, nous ne disposons que de très peu de références. Aussi nous proposons, une étude rétrospective, réalisée au service d’Endocrinologie de l’Hôpital Universitaire de Befelatanana et à la Clinique de l’Association Malgache contre le Diabète (AMADIA) à Faravohitra. Ceci a pour objectifs d’établir une gradation du risque podologique des diabétiques, de rechercher les facteurs associés ou aggravant ce risque, et d’évaluer les connaissances de ces patients concernant le risque podologique, le tout dans le but de réduire le taux d’amputation du pied diabétique.

## Méthodes

Il s’agissait une étude rétrospective descriptive et transversale, réalisée sur deux sites, sur une période de six mois, (1^er^ Février 2014 au 31 juillet 2014). Pour être inclus dans l’étude, les patients ont dû être diabétiques type 2 déjà connus ou nouvellement dépistés, hospitalisés, ou venant consulter auprès des deux sites, ne présentant aucune plaie ou ulcération au niveau des pieds au moment de l’examen. Le diagnostic d’un diabète nouvellement dépisté a été porté devant les critères classiques selon l’American Diabetes Association 2015 [1]. Nous avons exclu de notre étude les patients incapables de répondre aux questionnaires et n’ayant pas pu bénéficier des examens complémentaires requis pour la présente étude. L’âge, le genre, le secteur d’activité professionnelle du patient, l’ancienneté du diabète, le traitement antidiabétique actuel, les examens complémentaires faits dans les 12 derniers mois, les antécédents personnels, les complications majeures dégénératives du diabète ayant déjà existées, la connaissance du risque podologique par le patient, le type de chaussage habituel, la notion de déformation de(s) pied(s), l’existence d’une neuropathie périphérique et/ou artériopathie attestée par la méthode de l’International Consensus on the Diabetic Foot (2007) et le grade du risque podologique constituaient les paramètres de l’étude. Le risque podologique était classé selon la gradation du risque d’ulcération de l’International Working Group On Diabetic Foot (Tableau 1) [8]. Les données ont été recueillies à l’aide d’un questionnaire préétabli. Après chaque investigation une fiche d’éducation, a été remise aux patients selon le Consensus international sur le pied diabétique (IWGDF 2007). Pour l’exploitation des données, nous avons utilisé le Logiciel Epi Info (version 7.1.1.14) avec un test significatif p value = 0,5. Nous avons eu l’acceptation et l’autorisation de chaque chef hiérarchique des 2 centres. Tous les patients concernés par l’étude avaient consenti à y participer et avaient signé une fiche de consentement éclairé. Aucun conflit d’intérêt n’était à déclarer.

**Tableau 1:** Gradation du risque d’ulcération du pied chez les patients diabétiques selon l’International Working Group on Diabetic Foot

Grade	Définition	Risque de lésion
**0**	Absence de neuropathie sensitive	
**1**	Neuropathie sensitive isolée^+^	Multiplié par 5
**2**	Neuropathie sensitive associée à une artériopathie des membres inférieurs^++^ et/ou à une déformation du pied^+++^	Multiplié par 5 à 10
**3**	Antécédent d’ulcération du pied (grade 3a) et/ou d’amputation des membres inférieurs (grade 3b)	Multiplié par 25

+ Définie par l’anomalie au test au Monofilament de Semmes Weinstein (10g) ou un seuil de perception vibratoire > 25 V.

++ Définie par l’absence de pouls du pied ou un Index de Pression Systolique < 0,90

+++ Hallux valgus, orteils en marteau ou en griffe, proéminence de la tête des métatarsiens

## Résultats

**Figure 1:**
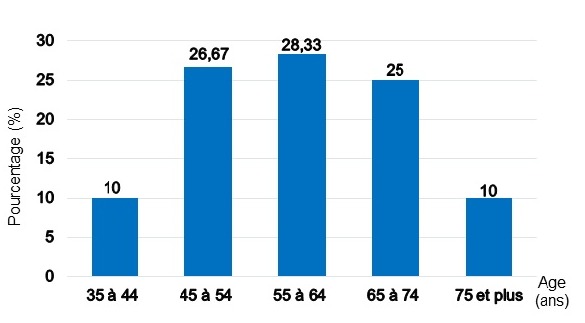
Répartition des patients selon l’âge

**Figure 2:**
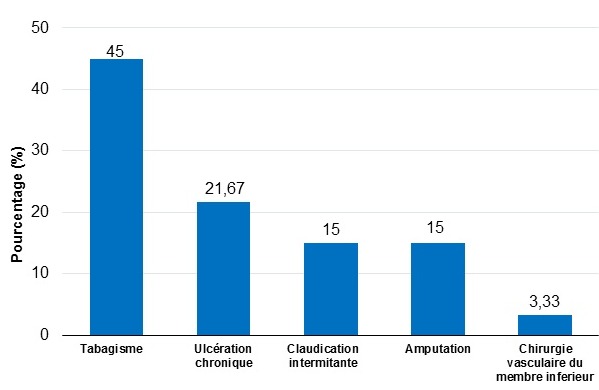
Répartition des patients selon les antécédents personnels

**Figure 3:**
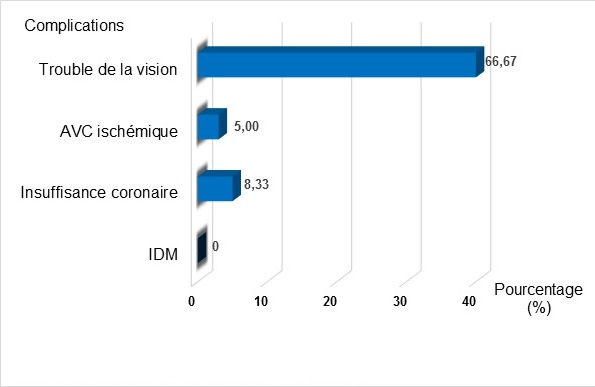
Répartition des patients selon les complications ayant déjà existé

**Figure 4:**
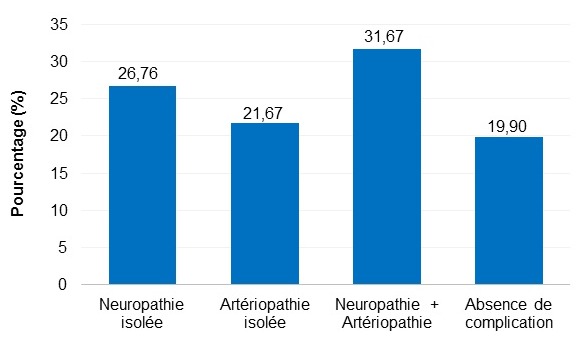
Répartition des patients selon les complications du pied

Pendant la période d’étude, beaucoup des patients diabétiques ont été hospitalisés et/ou vus en consultation externe à l’USFR en Endocrinologie et à l’AMADIA. Nous n’avons pas objectivé le nombre total des patients car sur le premier site, nous n’avons pas disposé de registre exact surtout pour les patients venant en consultation externe. Et nous n’avons retenu au total que 60 patients ayant répondu aux critères d’éligibilité. Il y avait 36 hommes et 24 femmes donnant un sex-ratio Homme/Femme de 1,5. Leur âge s’échelonnait de 38 à 87 ans. La moyenne d’âge était de 54,43 ans +/- 15,17 avec une majorité de sujets âgés entre 55 à 64 ans (Figure 1). Les retraités représentaient 33,33% de nos effectifs. Venaient ensuite, en terme de fréquence, les sujets à profession manuelle, puis les bureaucrates et les chômeurs. Aucun étudiant n’a été enregistré parmi notre population d’étude (Tableau 2). Le diabète a évolué entre 5 et 9 ans chez 36,67% de nos patients. Et la durée moyenne d’évolution du diabète a été de 7,35 ans +/- 6,58. Le tabagisme était l’antécédent le plus observé chez nos patients (45,00%), suivi des ulcérations chroniques du pied dans 20 % des cas. Un patient peut avoir un ou plusieurs antécédent(s) (Figure 2). Nous avons retrouvé une corrélation positive entre l’antécédent de tabagisme et le risque podologique (p ≤ 0,05). De même nous avons objectivé aussi une corrélation significative entre les antécédents d’ulcération chronique, l’amputation du membre inferieur et le risque podologique (p ≤ 0,05). Le trouble de la vision était la complication la plus représentée (66,67%), suivi de l’insuffisance coronarienne (8,33%). Certains patients ont déjà eu une ou plusieurs complication(s) (Figure 3). L’étude nous a permis de constater que 78,33% de nos patients avaient un diabète déséquilibré (Hb A1C > 7%), 73,33% avaient déjà une microalbuminurie ≥ 30mg / 24 heures et 76,67% une rétinopathie (Tableau 3). Elle a montré l’existence d’une corrélation positive entre le mauvais équilibre du diabète (Hb A1C) et le risque podologique (p ≤ 0,05). Parmi nos patients, 56,6% connaissaient le rôle du diabète dans les lésions du pied et 51,67% la conduite à tenir en cas de lésion (Tableau 4). Et 35% de nos patients marchaient encore pied nu, 30% portaient des chaussures serrés notamment les femmes. Au moment de l’examen, 20% de nos patients ont présenté une déformation du pied. Et nous avons retrouvé une corrélation significative entre cette déformation et le risque podologique (p ≤ 0,05). Parmi nos patients, 26,76% souffrent de neuropathie périphérique isolée et 31,67% de neuropathie associée à une artériopathie des membres inférieurs (Figure 4). Parmi notre population d’étude, 22% présentent un risque podologique de grade 3, 32% de grade 2,26% de grade 1 et 26% de grade 0.

**Tableau 2:** Répartition des patients selon le secteur d’activité professionnelle

Profession	Manuelle	Bureaucrate	Chômeur	Retraité	Etudiant
Nombre	16	12	12	20	0
Pourcentage (%)	26,67	20,00	20,00	33,33	0,00

**Tableau 3:** Répartition des patients selon les examens paracliniques faits dans les douze derniers mois et leurs résultats

Examens paracliniques faits dans les 12 derniers mois		Nombre de cas	Pourcentage (%)
Hb A1C	≤ 7 %	13	21,67
	> 7 %	47	78,33
Microalbuminurie	< 30 mg / 24h	16	26,67
	≥ 30 mg / 24h	44	73,33
Fond d'œil	Pas de rétinopathie	14	23,33
	Rétinopathie	46	76,67
ECG de repos	Normal	44	73,33
	Anormal	16	26,67

**Tableau 4:** Répartition des patients selon la connaissance du risque podologique

	Nombre de cas	Pourcentage (%)
**Pourquoi faut-il faire attention à ses pieds?**		
A cause du diabète	34	56,6
Autre raison	26	43,33
**Avez-vous l’habitude d’examiner vos pieds?**		
Oui	26	43,33
Non	34	56,67
**Avez-vous l’habitude de prendre soins de vos pieds?**		
Oui	27	45,00
Non	33	55,00
Que faire en cas de lésion au pied?		
Consulter son médecin traitant	31	51,67
Autre réponse	29	48,33

## Discussion

Notre étude était limitée par la taille de l’échantillon. Ce qui s’expliquait, par le fait que, l’examen a été fait par un seul Médecin. Aussi les résultats que nous avons obtenus ne sont pas extrapolables à toute la population diabétique à Madagascar. Nous n’avons pas disposé d’un diapason médical pour faire le test vibratoire dans la recherche de neuropathie périphérique. Or, Reilhes et al ont conclu que le seuil de perception vibratoire identifie beaucoup plus les patients à risque [9]. Pour évaluer l’Index de Pression Systolique dans la recherche d’une artériopathie, nous n’avons pas disposé d’un Doppler portatif. Ce qui pourrait sous-estimer la fréquence d’artériopathie dans la population d’étude.

Dans notre population d’étude, nous avons trouvé une prédominance de sexe masculin avec un sex-ratio de 1,5. Ce qui se rapproche de la répartition de la population d’étude de Noiry et al qui a retrouvé un sex-ratio de 1,12 [10], et de l’étude ENTRED 2007-2010 avec prédominance du sexe masculin à 54 % [6]. Nous n’avons pas retrouvé une corrélation significative entre le genre et le risque podologique dans notre étude. Cependant, dans l’étude cas-contrôlé de Lavery, le sexe masculin est un facteur de risque significatif d’ulcération en analyse multivariée [11]. Ceci pourrait être dû à la faible taille de notre échantillon. L’âge moyen de nos patients a été de 54,43 ± 15,17 ans. Ceci se rapproche celle de l’étude de Monabeka et al qui est de 54,6 ans ± 11,4 [12]. L’âge de nos patients n’a pas influencé le risque podologique (p = 0,90). Mais on constate que plus l’âge des patients avance, plus leur risque podologique se situe dans le grade 2 ou 3. Selon la littérature, les patients diabétiques âgés sont particulièrement affectés par les complications au niveau du pied [13]. En effet, les sujets plus âgés ont probablement une durée d’évolution plus longue de diabète. Donc il serait plus probable qu’ils aient développé plus de complications dégénératives du diabète, y compris l’ulcération du pied. La profession est considérée comme un des facteurs majeurs déterminant le niveau socio-économique d’une population. Il semblerait, d’après notre étude, que les populations les plus vulnérables sur le plan économique et financier soient les plus à risque d’ulcération du pied. Il s’agissait des retraités et de ceux travaillant dans le secteur manuel ainsi que des chômeurs. Ce constat rejoint celui de la littérature. La prévalence du diabète de type 2 est plus importante dans la population au statut socioprofessionnel moins favorisé. Les complications y sont plus importantes et la prise en charge moins bonne [14]. Dans notre étude, la durée moyenne de l’évolution du diabète était de 7,35 ans. Ce qui était inférieur à celle retrouvée par l’étude ENTRED 2007-2010, laquelle était de 11 ans [6].

Cette différence pourrait s’expliquer par le retard fréquent de diagnostic du diabète lié aux difficultés rencontrées pour un dépistage systématique du diabète dans les pays à faible revenus comme le nôtre. De notre côté, nous n’avons pas retrouvé une corrélation significative entre l’ancienneté du diabète et le risque podologique. Or, selon plusieurs études, la durée d’évolution du diabète est en général considérée comme un facteur de risque d’ulcération [3,15]. Ceci est probablement lié à la taille de notre échantillon. Dans notre étude, nous avons constaté que 45,00% de nos patients étaient tabagiques actifs avec une corrélation significative. Ceci rejoint celle des plusieurs études attestant que le tabac constitue un facteur de risque indépendant prédictif d’une ulcération du pied [16,17]. Parmi nos patients, 20% avaient déjà eu une ulcération chronique du pied. Ce qui était largement supérieur au pourcentage rapporté par Noiry (4,9% d’antécédents d’ulcération chronique du pied) [10]. Nous avons objectivée une corrélation significative (p ≤ 0,05) entre antécédent d’ulcère chronique du pied et risque podologique. Des auteurs ont confirmé que ceci constitue un facteur prédisposant à une amputation chez les diabétique [18,19]. Dans notre série, huit patients (13,33%) ont déjà été amputés au niveau des membres inférieurs. Dans les séries Africaines, le taux d’amputation tourne aux alentours de 46,3 à 50% des cas [19].

L’impact de ces antécédents sur la survenue d’une ulcération s’explique vraisemblablement par la persistance des facteurs délétères ayant abouti à la survenue des ulcérations antérieures. En outre, l’amputation entraîne des modifications biomécaniques et structurales augmentant le risque d’une future ulcération [3]. En ce qui concerne les antécédents d’artériopathie, 15% de nos patients ont déjà un antécédent de claudication intermittente. En effet, la claudication intermittente, qui représente le principal signe subjectif d’artériopathie des membres inférieurs, est souvent absente chez les diabétiques du fait de la neuropathie associée [20]. Dans notre série, 8,33% de nos patients souffraient déjà d’insuffisance coronaire, 5% d’Accident Vasculaire Cérébral (AVC) et 66,7% de trouble de la vision. Zamouri et al ont retrouvé de leur côté les antécédents d’insuffisance coronaire chez 12% de leurs patients, d’AVC dans 0% cas et de cécité dans 2,5 % des cas [21]. Seuls 21,67% de nos patients avaient eu un taux Hb A1C optimale. Ce qui est loin des résultats de l’ENTRED 2007-2010 avec 35% d’Hb A1C inférieur à 6,5% dans leur population d’étude [6]. Il reste donc à optimiser la prise en charge d’une grande majorité de nos patients diabétiques pour atteindre les objectifs recommandés par l’ADA. Concernant notre étude, il y avait une corrélation entre le mauvais équilibre du diabète et le risque podologique. De même, dans la littérature, un taux initial élevé d’HbA1c est associé à un risque accru d’ulcération [3,22]. Toute augmentation de l’HbA1c de 1% s’accompagne d’une augmentation de 28% du risque d’artériopathie des membres inférieurs à 6 ans [23]. Nos résultats ont retrouvé une population de 73,33% de patients présentant une microalbuminurie ≥ 30 mg/l en faveur d’une néphropathie diabétique. Hamonet et coll, dans leur étude, ont retrouvé que 25,6% de leurs patients présentaient déjà une néphropathie diabétique tout stade confondu [24].

Ceci pourrait s’expliquer par le retard de dépistage du diabète dans les pays à faible revenu comme Madagascar, et aussi de la mauvaise prise en charge de la maladie au sein de la population défavorisée comme la nôtre. Enfin, nos diabétiques sont diagnostiqués à la phase multi compliquée y compris l’insuffisance rénale. Ceci nous a permis ainsi de souligner l’insuffisance de dépistage précoce et de prise en charge des complications dégénératives du diabète dans notre pays, bien qu’il s’agisse d’une étude faite auprès des 2 centres spécialisés de référence en la matière. Il reste donc un grand effort à faire pour suivre les recommandations de l’ADA ou de la HAS concernant le suivi du diabète dans les pays à faible revenu comme le nôtre. Parmi nos patients, 56,6% connaissaient le rôle néfaste du diabète dans la survenue des lésions du pied et plus de la moitié d’entre eux ne prenaient pas soin de leurs pieds. Ces résultats vont dans le même sens qu’une enquête menée par Morrachini. Elle montrait que 94 % des patients étaient conscients du risque mais moins de 20 % donnaient des exemples précis sur les conduites adaptées [25]. Nous avons pu constater que l’éducation de nos patients et de leurs familles telle l’inspection quotidienne des pieds, les soins podologiques réguliers,… est encore loin d’être suffisant comme l’Internationale Diabetes Federation l’a recommandé [26]. Ceci reste donc un grand défi à faire dans notre pratique quotidienne. Ainsi, nous suggérons que tous les diabétiques soient éduqués et informés sur les mesures à prendre pour prévenir les lésions du pied. Vingt et un (soit 35%) de nos patients marchaient encore pied nu et 18 (soit 30,00%) portaient des chaussures serrées. Dans une série Européenne, 18,3% des patients déclaraient éviter de marcher pieds nus, 23,2% faisaient attention à la qualité de leur chaussage [10]. Nos résultats pourraient s’expliquer par la méconnaissance du risque par nos patients et par leur situation financière. Le choix des chaussures est aussi très important. Mais cette recommandation est encore difficile à réaliser chez nous. Nous n’avons que deux Centres d’Appareillage pour fabriquer des chaussures orthopédiques adéquates à Madagascar. De plus, les diabétiques à faible revenu, qui étaient majoritaires, sont limités par leurs moyens financiers.

Selon la littérature, les déformations du pied sont la source des zones d’hyperpression et de conflits accentuées en particulier avec le chaussage, à l’origine d’une ulcération ou même d’amputation [27]. Ces déformations constituent donc des facteurs de risque aggravants. Ceci était objectivé dans notre étude. L’examen clinique nous ont permis de diagnostiquer une neuropathie périphérique isolée dans 21,67% de cas, une artériopathie isolée dans 21,67 % et une association neuropathie-artériopathie dans 31,67%. Ces résultats se rapprochent de ceux retrouvés par Hamonet et son équipe dont les patients présentaient un diabète compliqué de: 17,9% de neuropathie isolée, 12,8 % d’artériopathie isolée [24]. Les neuropathies périphériques, sensitivomotrices et autonomes, sont fréquente et représentent les complications à l’origine des lésions du pied diabétique, avec perte d’alerte douloureuse, déformations du pied, hyperappui et sécheresse cutanée [28]. En plus l’artériopathie est un facteur d’aggravation très important responsable de retard de cicatrisation et de gangrène à l’origine fréquente d’amputation [28]. Ainsi l’examen clinque focalisé sur les membres inférieurs des diabétiques est fortement recommandé. D’où l’intérêt de grader le risque podologique de façon annuelle au minimum et de promouvoir la multidisciplinarité dans la prise en charge du pied diabétique. Le système de classification de « l’IWGF 2007 » nous a permis d’objectiver que 54% de nos patients avaient un risque podologique élevé (grade 2 et 3). De même en Guinée, dans l’initiative DAFI, 91% des patients avaient un risque élevé [29].

## Conclusion

Le pied diabétique représente une part importante des dépenses de santé liées au diabète. Ce qui fait de leur prévention un point clé incontournable. Il ne peut y avoir de stratégie de prévention efficace sans un dépistage précoce et une identification de la population à risque. Ce dépistage passe par le recueil des facteurs de risque et plus particulièrement par la recherche d’une neuropathie à l’aide du Monofilament ou d’un autre outil validé. L’examen des pieds doit constituer une rubrique obligatoire au cours de toute consultation de routine. Dans notre étude, nous avons constaté que le risque podologique de nos patients est très élevé. Il concerne surtout les couches les plus vulnérables sur le plan social: sujets relativement âgés et ayant un niveau socio-économique bas. Nous avons retenu comme facteurs prédictifs d’ulcération: le tabagisme, l’antécédent d’ulcération chronique des membres inférieurs, le mauvais équilibre du diabète et l’existence d’une déformation du pied. Ce qui implique donc l’arrêt du tabac pour les diabétiques. Il faut avoir un équilibre glycémique optimal. Quel que soit leur risque podologique, beaucoup de patients ne connaissent pas ce risque et les conduites à tenir adéquates face à ce risque. Il convient alors d’éduquer, tous les patients diabétiques et leur famille à chaque consultation. A la fin de chaque investigation, nous avons remis une fiche d’éducation pour chaque patient. Notre étude a recruté des patients ayant des complications multiples et des grades de risque podologique élevé.

### Etat des connaissances actuelles sur le sujet

- Les facteurs associés ou aggravant du risque podologique des diabétiques sont l’âge moyen du patient, la durée moyenne de l’évolution du diabète, le tabac, les antécédents d’artériopathie, d’ulcération chronique et d’amputation du pied, le mauvais équilibre du diabète et la déformation du pied;
- Le dépistage du risque podologique est fortement recommandé chez les diabétiques.

### Contribution de notre étude à la connaissance

- Les facteurs prédictifs d’ulcération identifiée étaient le tabagisme, l’antécédent d’ulcération chronique des membres inférieurs, le mauvais équilibre du diabète et l’existence d’une déformation du pied;
- Beaucoup de patients ne connaissent pas ce risque et les conduites à tenir adéquates face à ce risque;
- Les Médecins traitants négligent ou même ignorent ce risque.

## Conflits d’intérêts

Les auteurs ne déclarent aucun conflit d'intérêt.
